# Maternal Deprivation in Rats Decreases the Expression of Interneuron Markers in the Neocortex and Hippocampus

**DOI:** 10.3389/fnana.2021.670766

**Published:** 2021-06-08

**Authors:** Milan Aksic, Joko Poleksic, Dubravka Aleksic, Natasa Petronijevic, Nevena V. Radonjic, Maja Jakovcevski, Slobodan Kapor, Nevena Divac, Branislav R. Filipovic, Igor Jakovcevski

**Affiliations:** ^1^Institute of Anatomy Niko Miljanić, School of Medicine, University of Belgrade, Belgrade, Serbia; ^2^Institute of Medical and Clinical Biochemistry, School of Medicine, University of Belgrade, Belgrade, Serbia; ^3^Department of Psychiatry, University of Connecticut School of Medicine, Farmington, CT, United States; ^4^Department of Anatomy, Faculty of Medical Sciences, University of Kragujevac, Kragujevac, Serbia; ^5^Department of Pharmacology, Clinical Pharmacology and Toxicology, School of Medicine, University of Belgrade, Belgrade, Serbia; ^6^Abteilung für Neuroanatomie und Molekulare Hirnforschung, Ruhr-Universität Bochum, Bochum, Germany; ^7^Institut für Anatomie und Klinische Morphologie, Universität Witten/Herdecke, Witten, Germany

**Keywords:** cerebral cortex, hippocampus, interneurons, maternal deprivation, schizophrenia, synapses

## Abstract

Early life stress has profound effects on the development of the central nervous system. We exposed 9-day-old rat pups to a 24 h maternal deprivation (MD) and sacrificed them as young adults (60-day-old), with the aim to study the effects of early stress on forebrain circuitry. We estimated numbers of various immunohistochemically defined interneuron subpopulations in several neocortical regions and in the hippocampus. MD rats showed reduced numbers of parvalbumin-expressing interneurons in the CA1 region of the hippocampus and in the prefrontal cortex, compared with controls. Numbers of reelin-expressing and calretinin-expressing interneurons were also reduced in the CA1 and CA3 hippocampal areas, but unaltered in the neocortex of MD rats. The number of calbinin-expressing interneurons in the neocortex was similar in the MD rats compared with controls. We analyzed cell death in 15-day-old rats after MD and found no difference compared to control rats. Thus, our results more likely reflect the downregulation of markers than the actual loss of interneurons. To investigate synaptic activity in the hippocampus we immunostained for glutamatergic and inhibitory vesicular transporters. The number of inhibitory synapses was decreased in the CA1 and CA3 regions of the hippocampus in MD rats, with the normal number of excitatory synapses. Our results indicate complex, cell type-specific, and region-specific alterations in the inhibitory circuitry induced by maternal deprivation. Such alterations may underlie symptoms of MD at the behavioral level and possibly contribute to mechanisms by which early life stress causes neuropsychiatric disorders, such as schizophrenia.

## Introduction

According to the neurodevelopmental theory of schizophrenia, this mental illness represents the end stage of a process that starts long before its clinical presentation and is caused by the combination of early life stress and genetic factors (Kinros et al., 2010; Rapoport et al., 2012; Bora, 2015). To date, a growing body of evidence points out the relationship between traumatic or highly stressful situations in early life and vulnerability to mental illness (Brown and Derkits, 2010; Brietzke et al., 2012). Animal models of early life stress are a widely used research tool for investigating the linkage between early life adversities and psychopathologies with the onset in late adolescence or young adulthood such as schizophrenia (Ellenbroek et al., 1998; Lim et al., 2011; Stamatakis et al., 2014; Mhillaj et al., 2015). Maternal deprivation (MD) in rats, performed at postnatal day (P) 9 for 24 h, leads to the increase of circulating corticosterone during the “stress hyporesponsive period” thus promoting the neurodegenarative effects of glucocorticoids (Levine, 2001; Viveros et al., 2010). Three types of stressors underlie extensive glucocorticoid release: (1) the lack of maternal care during 24 h; (2) the lack of nutrients and (3) hypothermia—following the immature thermal regulatory system in neonate pups (Marco et al., 2015). Also, MD has long–term consequences such as impairment in declarative memory (Llorente et al., 2011), sensorimotor gating (Ellenbroek et al., 1998, 2004; Husum et al., 2002), synapse formation and stabilization (Choy et al., 2008; Marco et al., 2013), decreased numbers of NeuN-expressing neurons in the retrosplenial and prefrontal cortex, amygdala and nucleus accumbens (Aksić et al., 2013; Aleksić et al., 2016). We used the P9 maternal deprivation model, as it has shown the strongest effects on behavior (Ellenbroek et al., 1998) when compared with earlier stress (at days 3 or 6). Additionally, this model has shown a strong impact on increasing oxidative stress in the brains of affected animals (Marković et al., 2017). Taken together, this MD protocol could be used to model some pathological features of schizophrenia.

Although they represent a minority of the total neuron population in the brain, gamma aminobutyric acid (GABA)-ergic interneurons are crucial for the establishment of excitatory/inhibitory balance and for the fine-tuning of neuronal circuitry (Marín, 2012; Kepecs and Fishell, 2014). Multiple attempts to classify cortical and hippocampal interneurons have identified many morphologically, physiologically, and molecularly distinct subclasses (Ascoli et al., 2008). During the past decade, a growing amount of data indicates the presence of abnormal inhibitory function in patients with schizophrenia accompanied by altered synthesis and reuptake of GABA (Uhlhaas and Singer, 2010; Chen et al., 2014). Postmortem studies on schizophrenic patients reveal decreased glutamate decarboxylase 67 gene expression and protein levels as well as reduced GABA transporter 1 in the prefrontal cortex (Schleimer et al., 2004; Hashimoto et al., 2008; Curley et al., 2011; Kimoto et al., 2014). These changes seem to be predominantly present in the parvalbumin-expressing (PV+) interneurons, thought to be of major importance in suppressing pyramidal neuron firing driven by inputs conveying irrelevant information (Hashimoto et al., 2003; Uhlhaas and Singer, 2010). The numerical density of PV+ interneurons and/or PV fluorescence intensity was reported in the hippocampus and prefrontal cortex of the schizophrenic patients (Reynolds et al., 2002; Zhang and Reynolds, 2002; Konradi et al., 2011; Enwright et al., 2016). Furthermore, PV-expressing fast-spiking interneurons are of critical importance for the generation and maintenance of gamma rhythms in the hippocampus (Allen and Monyer, 2015; Bocker et al., 2019). However, studies on calbindin (CB)+ and calretinin (CR)+ interneurons in schizophrenic patients have yielded conflicting results (Brisch et al., 2015). Reelin, a protein implicated in neuronal migration and synapse formation, is expressed in a subclass of GABAergic neurons in the postnatal brain (Ishii et al., 2016). In schizophrenic patients, reelin is found to be expressed to a lesser degree in the hippocampus and prefrontal cortex compared to the healthy controls (Eastwood and Harrison, 2006). Previous studies that implemented chronic MD protocol (3–4 h/day) reported the reduction in PV expression and cell densities in the prefrontal cortex (Leussis et al., 2012; Do Prado et al., 2016), as well as increased CB and CR immunoreactivity in the hippocampus of the stressed animals (Giachino et al., 2007).

The aim of our study was to determine the long–term effects of early acute MD on numbers of parvalbumin, calbindin, calretinin, and reelin expressing interneurons in the neocortex and hippocampus. Additionally, to understand the impact of alterations in interneuron populations on the synaptic transmission, we examined the expression of inhibitory and excitatory vesicular transporters in the hippocampus.

## Materials and Methods

### Animal Care and Maternal Deprivation Protocol

Four male and eight nulliparous female Wistar rats, 3-month-old, were put together in the standard plexiglass cages with sawdust (26 × 42 × 15 cm), in a temperature (23 ± 1°C) and humidity (40–70%) controlled facility. The animals were maintained in a standard 12 h light/dark cycle (lights on at 07:00 am), with water and food available *ad libitum*. After 14 days dams were isolated and checked twice a day for delivery. The day of delivery was denoted as P0. On P9, half of the litters were subjected to the MD procedure according to the previously described protocol (Ellenbroek et al., 1998; Roceri et al., 2002). In brief, for MD group dams were removed from the litter at 10:00 am, after which the pups were weighed and placed back in their home cage where they remained undisturbed until the next day when at 10:00 am the dams were returned to their corresponding home cage. Control litters were only subjected to a brief (3 min) separation at P9 when pups were weighed. All litters were later left undisturbed except for the routine cleaning of the cages. On P22, animals were weaned and housed in the same sex, same group (MD, Control) of three to four animals per cage. Animals were sacrificed at P60, as young adults. Overall, 10 animals per group from four litters were used for this study: six animals per group were used for histological and four animals per group for biochemical experiments. Another group of two male and four female adult rats was put together and the experimental procedure repeated as described above, with one difference: at 15 days of age, 6 days after MD pups were sacrificed to check for cell death. For this experiment, four animals per group were used, out of two litters. All efforts were made to minimize animal suffering and reduce the number of animals used in the study. All experiments were carried out according to the NIH Guide for Care and Use of Laboratory Animals and were approved by the Ethics Committee of the University of Belgrade (permit number 2014-05/2).

### Tissue Processing for Histology

For the immunohistochemistry, at P15 or P60, young adult MD and control rats (*n* = 6/group) were anesthetized with chloral hydrate (3 mg/kg, i.p.) and transcardially perfused with fixative (4% formaldehyde in 0.1 M phosphate buffer) solution. Following decapitation, brains were extracted, post-fixed for 24 h at +4°C and cryoprotected by infiltration with sucrose for 2 days at 4°C (20% sucrose in 0.1 M phosphate buffer). The brains were frozen by immersion in 2-methylbutane (Sigma–Aldrich, St. Louis, MO) precooled to −80°C, and stored at −80°C until cutting. Serial coronal sections of 25 μm in thickness were cut on a freezing cryostat (Leica Instruments, Nußloch, Germany) at −25°C, collected on SuperFrost Plus glass slides (Menzel, Braunschweig, Germany) in a spaced serial sequence (four sections 250 μm apart were present on each slide) and stored at −20°C until use.

### Immunohistochemistry

For immunofluorescence staining, antigen retrieval procedure was performed in 0.01 M sodium citrate solution, pH 9.0, for 30 min at 80°C in a water bath. Nonspecific binding was blocked using 5% normal goat serum dissolved in phosphate-buffered saline (PBS), pH 7.3 and supplemented with 0.2% Triton X-100, 0.02% sodium azide for 1 h at room temperature. Table 1 lists primary antibodies used for immunohistochemical studies. The primary antibodies were diluted in PBS (pH 7.3) containing 0.5% lambda-carrageenan (Sigma) and 0.2% sodium azide and applied to the sections for 2 days at 4 °C. Following three washes for 15 min in PBS, the sections were incubated for 2 h at room temperature with the appropriate goat Alexa 488-conjugated secondary antibodies diluted at 1:200 in PBS. After two subsequent washes in PBS, nuclear counterstaining was performed using 4,6-diamidino-2 phenylindole (DAPI, 1:4,000, Molecular Probes, Eugene, OR, USA) for 10 min. Slices were again washed in PBS, mounted in Vectashield anti-fade medium (Biozol, Echig, Germany), and left to dry for 24 h before analysis. The specificity of immunostaining was controlled by replacing the primary antibody with the normal goat serum, which lead to the absence of staining.

**Table 1:** Antibodies used in this study.

**Antigen**	**Host**	**Dilution**	**Manufacturer**	**Catalog no.**
Parvalbumin	monoclonal mouse	1:1,000	Sigma–Aldrich	PARV-19
Calbindin	monoclonal rabbit	1:1,000	Sigma–Aldrich	C9848
Calretinin	polyclonal mouse	1:1,000	Sigma–Aldrich	269A-1
Reelin	monoclonal rabbit	1:500	Merck Millipore	MAB 5366
Activated caspase 3	polyclonal mouse	1:100	Santa Cruz	SC-7148
VGAT	monoclonal rabbit	1:1,000	Synaptic Systems	131011
VGLUT1	polyclonal mouse	1:1,000	Synaptic Systems	135303

### Fluorojade Staining for Cell Death

Sections were pre-warmed at 50 degrees for 30 min. They were washed in 1% NaOH solution in 80% ethanol for 5 min., then in 70% ethanol for 2 min, and distilled water for 2 min. Sections were then treated with 0.06% KMnO_4_ for 10 min, rinsed in distilled water for 2 min and incubated for 20 min in 0.0004% FluoroJade-B (Merck-Millipore) solution in 0.1% glacial acetic acid. Afterward, sections were rinsed 3 × 1 min in distilled water, dried overnight, dehydrated with xylene, and coverslipped as described above.

### Image Acquisition and Cell Counting

**Figure 1:**
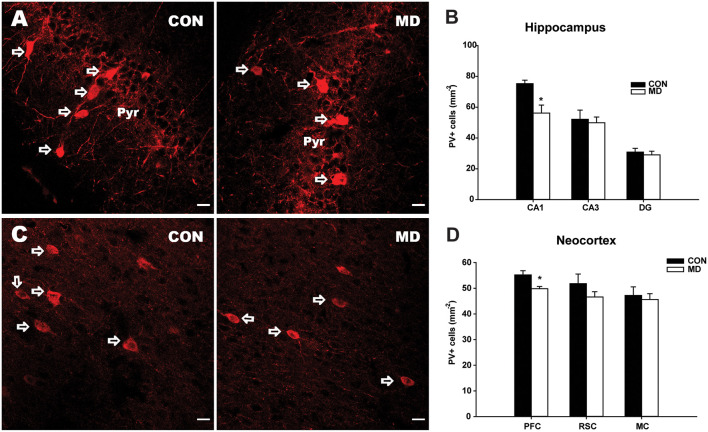
Parvalbumin-expressing interneurons. **(A)** Representative images of immunofluorescence staining for parvalbumin in the CA1 region of the hippocampus of a control (CON, left panel) and an maternal deprivation (MD; right panel) rat at P60. Pyr indicates a pyramidal cell layer. **(B)** Shown are mean values + standard error of the mean (SEM) for profile densities (number of immunopositive cells per area) of parvalbumin+ neurons in the hippocampus. **(C)** Representative images of immunofluorescence staining for parvalbumin in the prefrontal cortex of a CON (left panel) and an MD (right panel) rat at P60. **(D)** Shown are mean values + standard error of the mean (SEM) for profile densities (number of immunopositive cells per area) of parvalbumin+ neurons in the neocortex (*p < 0.05, *t*-test, *n* = 6 animals per group). Scale bars: 20 μm.

**Figure 2:**
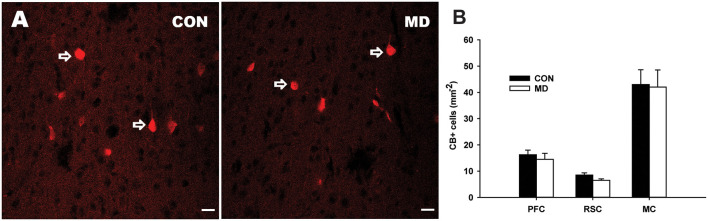
Calbindin-expressing neurons. **(A)** Representative images of immunofluorescence staining for calbindin in the prefrontal cortex of a control (CON, left panel) and MD (right panel) rat at P60. **(B)** Shown are mean values + standard error of the mean (SEM) for profile densities (number of immunopositive cells per area) of calbindin+ neurons in the neocortex of MD and CON rats (*p* > 0.05, *t*-test, *n* = 6 animals per group). Scale bars: 20 μm.

**Figure 3:**
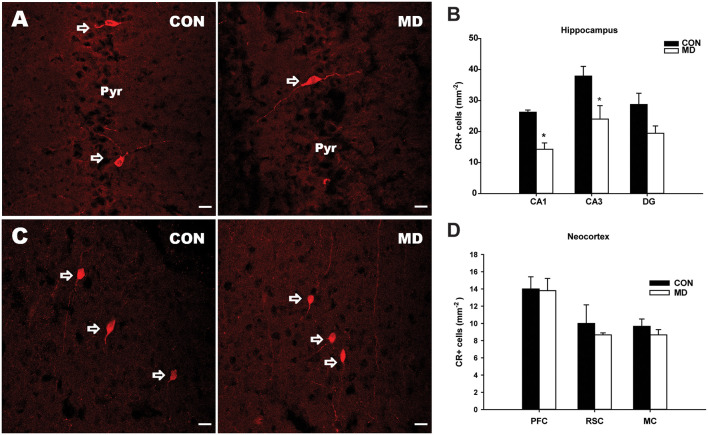
Calretinin-expressing interneurons. **(A)** Representative images of immunofluorescence staining for calretinin in the CA3 region of the hippocampus a control (CON, left panel) and an MD (right panel) rat at P60. Pyr indicates a pyramidal cell layer. **(B)** Shown are mean values + standard error of the mean (SEM) for profile densities (number of immunopositive cells per area) of calretinin+ neurons in the hippocampus of MD and control rats (*p < 0.05, *t*-test, *n* = 6 animals per group). **(C)** Representative images of immunofluorescence staining for calretinin in the retrosplenial cortex of a control (CON, left panel) and MD (right panel) rat at P60. **(D)** Shown are mean values + standard error of the mean (SEM) for profile densities (number of immunopositive cells per area) of calretinin+ neurons in the neocortex of MD and CON rats (*p* > 0.05, *t*-test, *n* = 6 animals per group). Scale bars: 20 μm.

**Figure 4:**
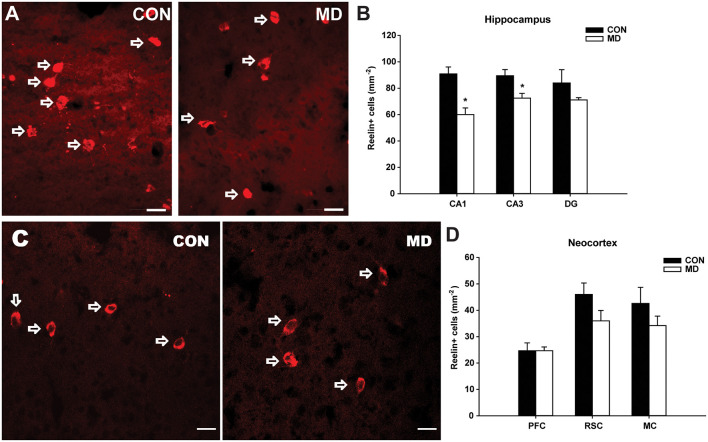
Reelin-expressing interneurons. **(A)** Representative images of immunofluorescence staining for reelin in the CA3 region of the hippocampus of a control (CON, left panel) and an MD rat at P60. **(B)** Shown are mean values + standard error of the mean (SEM) for profile densities (number of immunopositive cells per area) of reelin+ neurons in the hippocampus of MD and control rats (*p < 0.05, *t*-test, *n* = 6 animals per group). **(C)** Representative images of immunofluorescence staining for reelin in the retrosplenial cortex of a control (CON, left panel) and MD (right panel) rat at P60. **(D)** Shown are mean values + standard error of the mean (SEM) for profile densities (number of immunopositive cells per area) of reelin+ neurons in the neocortex of MD and CON rats (*p* > 0.05, *t*-test, *n* = 6 animals per group). Scale bars: 20 μm.

Image acquisition of brain sections was performed on an optical microscope (DM4000 Leica, Wetzlar, Germany) with a 40× objective and analyzed in Photoshop 7.0 software (Adobe, San Jose, CA), using a 1-cm rectangular grid. Previously, the anatomical delineations of the cortical and dorsal hippocampal regions (−2.40 to −3.72 mm distance from bregma) were defined by the nuclear staining pattern using ×10 objective according to the anatomical atlas (Paxinos and Watson, 2006). Pictures of whole areas were taken and cells immunoreactive for various interneuron markers were counted in spaced serial sections of rat brains at the same distance from bregma (2.52 mm for prefrontal cortex and −2.76 mm for retrosplenial and motor cortices). The criterion for the neuron to be counted was when the whole cell body was in focus on the image, as indicated by arrows on Figures 1–4. The counted numbers (density) of immunoreactive cells were expressed per unit area (mm^2^). At least 200 random microscope fields at 40× magnification oil immersion objective (area 53,056 μm^2^) out of five sections, from each of six animals per group were counted in the retrosplenial, motor cortex, and prefrontal cortex of each section. Left and right hippocampal and cortical areas were evaluated and, as no difference between the left and right hemispheres was detected, results were shown as averaged bilateral values. As the distribution of interneurons within cortical layers is another important parameter that defines interneuron subpopulations, we determined the percentage of immunostained cells within hippocampal strata, as well as in the cortex divided into upper (layers 2–3) and lower (layers 4–6) layers. For none of the markers tested here did the distribution significantly differ between control and maternally deprived rats, either in the hippocampus or in the cortical areas (data not shown).

### Synaptic Coverage

For quantification of glutamatergic and inhibitory transmission in the hippocampus, we used coronal sections of the dorsal hippocampus. Estimation of perisomatic inhibitory terminals around pyramidal/granular cell bodies was performed as described (Schmalbach et al., 2015). Briefly, stacks of 1-μm-thick images were obtained from sections stained for VGAT on an LSM 510 confocal microscope (Zeiss) using a 63× oil immersion objective with 1,024 × 1,024 pixel resolution. One image per cell at the level of the largest cell body cross-sectional area was used to measure the perimeter, as well as to count individually discernible perisomatic puncta. Numbers of vesicular inhibitory neurotransmitter transporter (VGAT)+ puncta were normalized to the perimeter of the cell profile (linear density). To analyze the intensity of vesicular glutamate transporter 1 (VGLUT1) immunostainings, pictures were taken from the areas labeled in Figure 6C using 20× objective at the same apertures and exposition times, using the same digital gain. To obtain an overall estimate of the mean pixel intensity (brightness range 55–255), entire image frames were quantified in the different experimental groups. The threshold was determined by measuring background brightness in the non-stained parts of the images. For data analysis, ImageJ freeware^1^ was used, as previously described (Vulovic et al., 2018).

**Figure 5:**
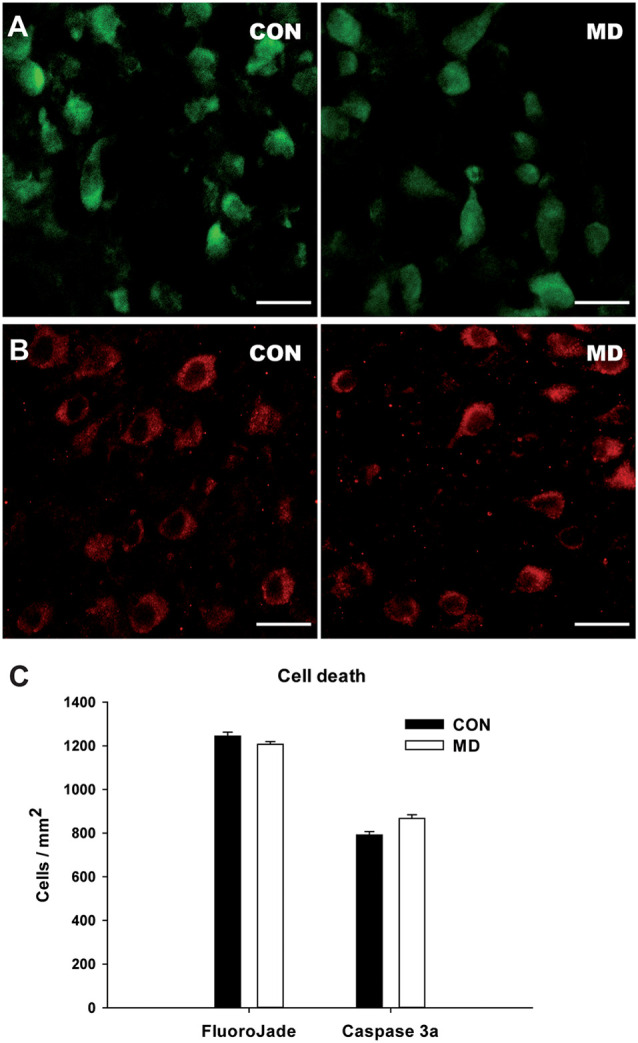
Cell death in the cortex at P15. **(A,B)** Representative images of the FlouroJade **(A)** and activated caspase 3 **(B)** stainings in the prefrontal cortex of P15 rats. Left panels show control (CON) and right panels MD rats. **(C)** Shown are mean values + standard error of the mean (SEM) for profile densities of labeled cells (*p* > 0.05, *t*-test, *n* = 4 animals per group). Scale bars: 20 μm.

**Figure 6:**
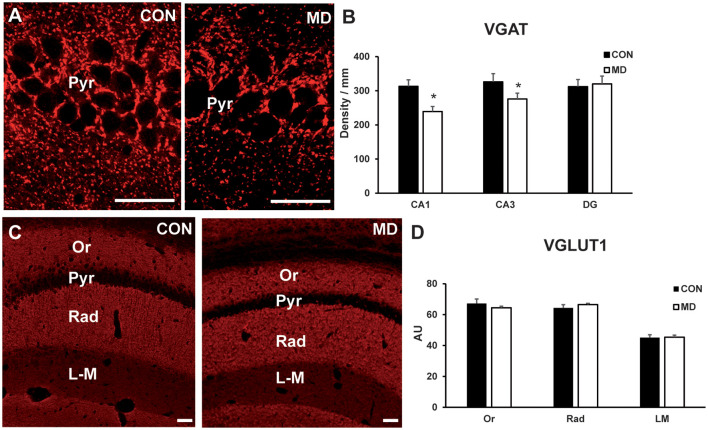
Inhibitory and excitatory synapses. **(A)** Representative image of immunofluorescence staining for VGAT in the CA1 region of the hippocampus of a control (CON, left panel) and maternally deprived (MD, right panel) rat at P60. **(B)** Shown are mean values + standard error of the mean (SEM) for linear densities (number of immunopositive puncta per unit length) of VGAT+ puncta in the pyramidal layers (Pyr) of the CA1 and CA3, and granular layer of dentate gyrus (DG) of MD and control rats (*p < 0.05, *t*-test, *n* = 6 animals per group). **(C)** Representative image of immunofluorescence staining for VGLUT1 in the hippocampus a control (CON, left panel) and maternally deprived (MD, right panel) rat at P60. **(D)** Shown are mean values + standard error of the mean (SEM) for mean fluorescence intensity of VGLUT1 in the stratum oriens (Or), radiatum (Rad) and lacunosum-moleculare (LM) of the CA1 hippocampal region of MD and CON rats (*p* > 0.05, *t*-test, *n* = 6 animals per group). Scale bars: 50 μm.

### Statistical Analysis

All the data have been taken into the database and processed by commercially available software. Besides standard measures of homogeneity (mean values, standard error of the mean—SEM), animal mean differences were compared using Student’s *t*-test, as our data had normal distribution and equal variance, as tested by Shapiro-Wilk and equal variance tests, respectively. All tests have been performed at 95% probability level.

## Results

### Parvalbumin-Expressing Interneurons

Maternal deprivation caused a statistically significant 25% reduction in density of PV+ cells in the CA1 (56.2 ± 5.2/mm^2^ vs. 75.29 ± 2.23/mm^2^ in MD vs. control rats, *n* = 6 animals/group, *t*-test, *p* = 0.009) subregion of the hippocampus, with no changes in the CA3 (49.9 ± 3.7/ mm^2^ vs. 52.16 ± 5.93/mm^2^ in MD vs. control rats, *n* = 6 animals/group, *t*-test, *p* = 0.74) and DG (29.01 ± 2.38/mm^2^ vs. 30.85 ± 2.38/mm^2^ in MD vs. control rats, *n* = 6 animals/group, *t*-test, *p* = 0.61) subregions, compared to control rats at P60 (Figures 1A,B). Furthermore, a small (10%), but significant reduction in density of PV+ cells was observed in the prefrontal cortex (49.8 ± 0.86/mm^2^ vs. 55.2 ± 1.62/mm^2^ in MD vs. control rats, *n* = 6 animals/group, *t*-test, *p* = 0.018), while no alterations were found in the retrosplenial (46.7 ± 2.06/mm^2^ vs. 51.8 ± 3.7/mm^2^ in MD vs. control rats, *n* = 6 animals/group, *t*-test, *p* = 0.25) and motor (45.6 ± 2.29/mm^2^ vs. 47.2 ± 3.32/mm^2^ in MD vs. control rats, *n* = 6 animals/group, *t*-test, *p* = 0.7) cortices (Figures 1C,D).

### Calbindin-Expressing Interneurons

Considering CB+ cells, none of the examined neocortical regions showed statistically significant differences between MD and control groups (Figure 2). The values for the prefrontal cortex were 14.5 ± 2.25/mm^2^ vs. 16.5 ± 1.74/mm^2^ in MD vs. control rats, *n* = 6 animals/group, *t*-test, *p* = 0.61, retrosplenial cortex 6.5 ± 0.56/mm^2^ vs. 8.5 ± 0.83/ mm^2^ in MD vs. control rats, *n* = 6 animals/group, *t*-test, *p* = 0.13, and motor cortex 42 ± 6.54/mm^2^ vs. 43 ± 5.62/mm^2^ in MD vs. control rats, *n* = 6 animals/group, *t*-test, *p* = 0.92. We did not quantify CB+ cells in the hippocampus, as it is expressed by both interneurons and principal cells in this region (Celio, 1990).

### Calretinin-Expressing Interneurons

The numbers of CR+ cells (Figure 3A) were significantly decreased in the CA1 (14.28 ± 2.04/mm^2^ vs. 26.25 ± 0.73/mm^2^ in MD vs. control rats, *n* = 6 animals/group, *t*-test, *p* = 0.005) and CA3 (24.05 ± 4.3/mm^2^ vs. 37.9 ± 3.1/mm^2^ in MD vs. control rats, *n* = 6 animals/group, *t*-test, *p* = 0.01) subregions of the hippocampus of MD rats when compared to the controls (Figure 3B), while no significant differences between the two groups were found in the DG (19.44 ± 2.38/mm^2^ vs. 28.75 ± 3.62/mm^2^ in MD vs. control rats, *n* = 6 animals/group, *t*-test, *p* = 0.09), although the same tendency was present (Figure 3B). Also, no significant differences in the number of CR+ cells were found in examined neocortical regions in MD group compared to the controls (Figures 3C,D). The values in prefrontal cortex were 13.8 ± 1.43/mm^2^ vs. 14 ± 1.41/mm^2^ in MD vs. control rats, *n* = 6 animals/group, *t*-test, *p* = 0.92, retrosplenial cortex 8.67 ± 0.23/ mm^2^ vs. 10 ± 1.08/mm^2^ in MD vs. control rats, *n* = 6 animals/group, *t*-test, *p* = 0.44, and motor cortex 8.67 ± 0.62/mm^2^ vs. 9.67 ± 0.85/mm^2^ in MD vs. control rats, *n* = 6 animals/group, *t*-test, *p* = 0.54.

### Reelin-Expressing Interneurons

Similar to calretinin, in the CA1 (60 ± 5.08/mm^2^ vs. 90.88 ± 5.19/mm^2^ in MD vs. control rats, *n* = 6 animals/group, *t*-test, *p* = 0.004) and CA3 (72.5 ± 3.59/ mm^2^ vs. 89.47 ± 4.65/mm^2^ in MD vs. control rats, *n* = 6 animals/group, *t*-test, *p* = 0.028) hippocampal subregions, a significant reduction in number of reelin+ interneurons (Figure 4A) was detected in MD group (Figure 4B). Congruently, no statistically significant differences were found neither in the DG (71.11 ± 1.71/mm^2^ vs. 84.03 ± 9.98/mm^2^ in MD vs. control rats, *n* = 6 animals/group, *t*-test, *p* = 0.29), nor in any of the examined neocortical areas between MD and control groups (Figure 4C). The values in prefrontal cortex were 24.65 ± 1.46/mm^2^ vs. 24.67 ± 2.97/mm^2^ in MD vs. control rats, *n* = 6 animals/group, *t*-test, *p* = 1, retrosplenial cortex 36 ± 3.94/mm^2^ vs. 46 ± 4.33/ mm^2^ in MD vs. control rats, *n* = 6 animals/group, *t*-test, *p* = 0.13, and motor cortex 34.2 ± 3.58/mm^2^ vs. 42.6 ± 6.07/mm^2^ in MD vs. control rats, *n* = 6 animals/group, *t*-test, *p* = 0.27.

### Cell Death in the Cortex at P15

To determine if our results reflect the actual loss of interneurons or the loss of marker proteins, we examined cell death at P15, 6 days after the maternal deprivation, at the time of the peak in physiological apoptotic cell death in the cortex (Schmid et al., 2013). We stained control and MD brain sections for FlouroJade (Figure 5A) and activated caspase 3 (Figure 5B), markers of neurodegeneration and apoptotic cell death. Using both markers, many cell were stained in both conditions (Figure 5), however, there was no difference in the number of stained cells between MD and control rats (Figure 5C). We thus conclude that our results are more likely to represent the decreased expression of markers than the actual loss of interneurons.

### Inhibitory and Excitatory Transmitters in the Hippocampus

To determine if there is an impact of the loss of interneuron markers on the circuitry, we immunostained rat brain sections for inhibitory presynaptic marker VGAT (Figure 6A), and excitatory presynaptic marker VGLUT1 (Figure 6C). The number of VGAT+ terminals per unit area (linear density) was lower in the CA1 pyramidal layer (313 ± 18.08/mm vs. 239.16 ± 15.03/mm in MD vs. control rats, *n* = 6 animals/group, *t*-test, *p* < 0.0001) and CA3 pyramidal layer (275.7 ± 23.4/ mm vs. 325.5 ± 18.8/mm in MD vs. control rats, *n* = 6 animals/group, *t*-test, *p* = 0.0022) regions of the hippocampus in MD rats compared to controls, whereas in the dentate gyrus granule cell layer (320.6 ± 23/mm vs. 312.5 ± 21/mm in MD vs. control rats, *n* = 6 animals/group, *t*-test, *p* = 0.54) values were similar for MD and control rats (Figure 6B). VGLUT1+ terminals were measured within the CA1 field, separately for stratum oriens, radiatum and lacunosum-moleculare (Figure 6C). For all these subregions, average values for the intensity of staining were similar between MD and control rats (Figure 6D). The values were in stratum oriens 63 ± 0.71 AU vs. 67.8 ± 3.25 AU in MD vs. control rats, *n* = 6 animals/group, *t*-test, *p* = 0.18; stratum radiatum 68.72 ± 0.72 AU vs. 63.8 ± 2.95 AU in MD vs. control rats, *n* = 6 animals/group, *t*-test, *p* = 0.14; and lacunosum-moleculare 47.9 ± 1.15 AU vs. 47.3 ± 2.25 AU in MD vs. control rats, *n* = 6 animals/group, *t*-test, *p* = 0.78. Therefore, maternal deprivation causes relative dominance of excitatory over inhibitory synapses.

## Discussion

In this study, we demonstrate an effect of early maternal deprivation on the decrease of marker expression by specific interneuron subpopulations in the hippocampus and prefrontal cortex. In addition, we show decreased VGAT expression in the hippocampal CA1 and CA3 regions of MD rats, coupled with normal VGLUT1 expression. In a previous study, we had found decreased total neuron population in the cerebral cortex of the young adult rats subjected to MD (Aksić et al., 2013). Because both, hippocampus and neocortex, abundantly express the glucocorticoid receptors (Ahima and Harlan, 1990; Van Eekelen and De Kloet, 1992; Ostrander et al., 2003), we hypothesized that acute MD, performed at P9 for 24 h, would cause harmful effects in examined interneuronal subclasses *via* extensive glucocorticoid release (Viveros et al., 2010; Xu et al., 2011).

During the neonatal period N-methyl-D-aspartate receptors (NMDAR) are of crucial importance for adequate structural and functional maturation of PV+ neurons and synaptic formation, consequently resulting in the development of mature GABAergic transmission (Matta et al., 2011). Early postnatal NMDAR dysfunction/ablation correlates with decreased numbers of PV+ interneurons, impairment of network synchrony, and cognitive symptoms including working memory loss (Belforte et al., 2010; Korotkova et al., 2010; Radonjić et al., 2013). As early life stress seems to disturb the physiological glutamate receptor subunits 2B/2A switch (Viviani et al., 2014) and alters NMDAR levels in the hippocampus and cortex (Roceri et al., 2002; Akillioglu et al., 2015; Manatos et al., 2016), we can speculate that NMDAR dysfunction during early postnatal development causes inadequate maturation of PV+ interneurons. In addition, an important event in development occurring between postnatal days 5 and 10 is switch in GABA action from excitatory to inhibitory, due to the switch in ion exchanger composition on the neuron cell membrane (Ben-Ari et al., 2007; Marguet et al., 2015). It is conceivable that stress during this vulnerable time in cortical circuitry development increases apoptotic cell death of supernumerous interneurons, which is also at its peak during the second week of postnatal development (Blomgren et al., 2007; Schmid et al., 2013). Furthermore, we cannot exclude the possibility that PV immunoreactivity loss is caused by oxidative stress or neuroinflammation (Francis and Stevenson, 2011; Holland et al., 2014; Schmalbach et al., 2015; Marković et al., 2017). Our finding of decreased parvalbumin expression is consistent with the results of earlier studies showing reduced PV expression and cell density in the prefrontal cortex of the adolescent, but not adult (P100) rats (Francis and Stevenson, 2011; Wieck et al., 2013; Holland et al., 2014; Grassi-Oliveira et al., 2016). However, in the hippocampus, Francis and Stevenson (2011) reported no change in PV expression, while in another study decreased PV+ neuron density was found in the DG subregion at weaning (Seidel et al., 2008). In our study, we demonstrated a reduction in the expression of calretinin in the CA1 and CA3 subregions of the hippocampus and no alterations in the examined cortical areas. Opposite findings were reported by other investigators, i.e., increased levels of calbindin and calretinin in the hippocampus of neonatal, peripubertal, and adolescent rats (Lephart and Watson, 1999; Giachino et al., 2007; Xu et al., 2011). We believe that difference reported in various studies are due to methodological difference in deprivation protocols and time points of examination.

During cortical development, reelin is secreted from the Cajal–Retzius cells in the marginal zone and plays a critical role in controlling neuronal migration and layer formation in the neocortex and hippocampus (D’Arcangelo et al., 1995). However, in the postnatal brain, reelin is predominantly expressed in the subpopulation of GABAergic interneurons and is involved in NMDA-mediated synaptic function, learning, and memory (Ishii et al., 2016). In this study we observed decreased numbers of reelin+ cells in the CA1 and CA3 subregions of the hippocampus, but not in the DG. Lower reelin mRNA and protein levels in the hippocampus of the peripubertal and young adult rats exposed to maternal deprivation procedures have been reported (Qin et al., 2011; Zhang et al., 2013). As glucocorticoid receptor expression is very high in the hippocampus, one possible explanation for these findings could be the deleterious effect of corticosterone during the early postnatal period (Fenton et al., 2015).

Our data show a reduction in several different interneuron populations in the hippocampus, as well as a reduction in parvalbumin-expressing neurons in the prefrontal cortex of MD rats compared to controls. As we could not detect increased cell death or signs of neurodegeneration in MD rats, the most parsimonious explanation is that the immunoreactivity to these markers is decreased in MD rats. This is particularly pertinent to parvalbumin-expressing neurons, which are known to downregulate parvalbumin expression upon oxidative stress and in schizophrenia patients (Akbarian et al., 1995; Schmalbach et al., 2015; Janickova and Schwaller, 2020). Our previous work has shown that MD rats have indeed increased levels of oxidative stress, as well as higher numbers of microglial cells in the hippocampus (Marković et al., 2017). Another possible interpretation of our results would be that degeneration and loss of interneurons occur at the later stage, between 15 and 60 days of age. However, it is possible to speculate that, regardless of whether the cells are lost or downregulate specific proteins, their function is impaired, as suggested by a decrease in VGAT immunoreactive synaptic terminals in MD rats.

In this manuscript, we have shown that perinatal MD induces alterations in the inhibitory circuitry during early adulthood, namely the loss of parvalbumin expression in the hippocampus CA1 region and prefrontal cortex, as well as reelin and calretinin expression and, importantly, VGAT+ synapses in the CA1 and CA3 hippocampus subfields. Hence, impaired excitatory/inhibitory balance in the hippocampus and neocortex may represent the underlying mechanism of cognitive impairment and sensory gating deficits, previously observed in this animal model (Ellenbroek et al., 2004; Marco et al., 2013). One of the weaknesses of our study was that we used only male rats, as we tried to avoid the possibility that change in sex hormones might influence variability. It has, however, been shown, using the same maternal deprivation model that behavioral outcome was similar between male and female rats (Ellenbroek et al., 1998). Further studies on maternally deprived animals throughout distinct periods of neurodevelopment are needed in order to examine the effects on the neural circuitry at the electrophysiological level.

## Data Availability Statement

The raw data supporting the conclusions of this article will be made available by the authors, without undue reservation.

## Ethics Statement

The animal study was reviewed and approved by Ethics Committee of the University of Belgrade.

## Author Contributions

NR, MA, and NP conceived the study. MA, JP, IJ, MJ, DA, and SK performed experiments and collected data. IJ, NR, BF, and MA designed experiments and data analysis. IJ, MA, and ND performed data analysis and wrote the manuscript. All authors contributed to the article and approved the submitted version.

## Conflict of Interest

The authors declare that the research was conducted in the absence of any commercial or financial relationships that could be construed as a potential conflict of interest.

## Footnotes

^1^https://imagej.nih.gov/ij/
